# Does a Widespread Species Have a Higher Competitive Ability Than an Endemic Species? A Case Study From the Dongting Lake Wetlands

**DOI:** 10.3389/fpls.2022.864316

**Published:** 2022-05-24

**Authors:** Yuhang Du, Qiaoqiao Zhou, Zenghui Peng, Fangcheng Peng, Lianlian Xi, Youzhi Li

**Affiliations:** ^1^College of Resources and Environment, Hunan Agricultural University, Changsha, China; ^2^Hunan Provincial Key Laboratory of Rural Ecosystem Health in Dongting Lake Area, Hunan Agricultural University, Changsha, China

**Keywords:** *Triarrhena lutarioriparia*, *Phragmites australis*, competition intensity, interspecific competition, relative growth rate, niche difference

## Abstract

The distribution range of plants is usually related to their competitiveness. The competitive ability between common widespread, which are generally considered to be invasive, and common endemic species, is still not very clear. Five plant communities were monitored in the field to compare the competitive abilities of widespread species, *Phragmites australis*, and endemic species, *Triarrhena lutarioriparia*, in the Dongting Lake wetlands. The ratios of individual numbers of *T. lutarioriparia* to *P. australis* per square meter were found to be 9:0, 14:1, 10:5, 7:6, and 0:11 in the five respective communities. A manipulation experiment was then performed with five planting modes (*T. lutarioriparia*: *P. australis* was 4:0, 3:1, 2:2, 1:3, and 0:4, respectively). Results from field monitoring showed that the two plant species exhibited similar decreased survival percentages during flooding. *P. australis* had higher aboveground biomass before the flooding and a higher relative elongation rate, whereas *T. lutarioriparia* had higher aboveground biomass after flooding and a higher relative growth rate (RGR). *P. australis* had a higher competitive ability than *T. lutarioriparia* before and after the flooding. The manipulation experiment revealed that *P. australis* had a higher survival percentage than *T. lutarioriparia*, with no differences in plant biomass, RGR, and the relative elongation rate between the two species. *P. australis* was found to have a higher competitive ability than *T. lutarioriparia* in the early growing stage and a lower competitive ability in the middle and later stages. The relative yield total in the field monitoring and manipulation experiment was 1, indicating that *T. lutarioriparia* and *P. australis* occupied different niches in the experimental conditions. It was concluded that, compared with *T. lutarioriparia*, *P. australis* has a higher competitive ability in submerged habitats and a lower competitive ability in the non-submerged habitat. The niche differences between the two species enabled their coexistence in the Dongting Lake wetlands with seasonal flooding.

## Introduction

Plants can be classified as widespread species and endemic species based on their distribution range (Hung et al., 2014; Bai et al., 2018). For example, *Stemona tuberosa*, a traditional medicinal plant, is distributed across more than ten countries in Asia (Chen et al., 2019), and the shrub *Menziesia pentandra* is distributed across Japan, Sakhalin, and the Southern Kuril Islands (Maki et al., 2002). *Cinnamomum kanehirae* is endemic to Taiwan at an elevation range of 200–2,000 m (Hung et al., 2014), and *Pilea carautae* is endemic to the Cabo Frio region in the State of Rio de Janeiro, Southern Brazil (Filho and Alves, 2010). The distribution of a plant is associated with its competitive ability, as it reflects its ability to obtain resources such as light, water, and nutrients, as well as its tolerance to resource stress (Schob et al., 2013). Therefore, widespread plant species appear to have higher competitive abilities than endemic plant species (Zhang and van Kleunen, 2019).

Plant competition includes intraspecific and interspecific competition and is determined by the competition intensity, effects, and outcomes (Connolly, 1987; Weigelt and Jolliffe, 2003). Competitive effects have been used to analyze and predict changes in plant populations and community structure (Goldberg and Werner, 1983; Miller and Werner, 1987; Pan et al., 2021). Under the conditions of water and nitrogen deficiency, the higher competitive ability of the invasive plant *Xanthium italicum* compared with the native plant *Xanthium sibiricum* was the main reason for the succession of *X. sibiricum* by *X. italicum* in its native habitat (Wang, 2018). With an increase in nitrogen levels, *Suaeda salsa* was found to have a higher competitive ability than a congeneric species *Suaeda glauca*, with *S. salsa* gradually dominating the mixed communities (Qi, 2019). Four meta-analyses of hundreds of experiments showed that widespread plant species are more competitive than endemic species (Vilà et al., 2011; Kuebbing and Nunez, 2016; Golivets and Wallin, 2018; Zhang and van Kleunen, 2019). However, the results of these studies may be biased for using common, often invasive, widespread species (Hulme et al., 2013) and rare endemic species (Vilà and Weiner, 2004). It is essential to test the competitive ability between common widespread species and common endemic species to gain a comprehensive understanding of the determinants of invasion success (Maki et al., 2002; Conesa et al., 2008).

*Phragmites australis* is a widespread species that is commonly distributed across North America (Altartouri et al., 2015; Eller et al., 2017), Europe (Van der Putten, 1997; Klimes, 2000), and Asia (Clevering and Lissner, 1999; Li et al., 2011). *Triarrhena lutarioriparia* is an endemic species that is only distributed along the middle and lower reaches of the Yangtze River, China (Liu et al., 2001; Wang et al., 2019). These two plant species are dominant species in the Dongting Lake, the second-largest freshwater lake in China, and are distributed at the same altitude (24.55–25.10 m above sea level) on the beaches of the lake (Zhuang et al., 2010; Li et al., 2016). These plant species have similar morphological, growth, and propagation characteristics. For example, the specific leaf area of *P. australis* was 280–290 and 290–300 cm^2^ g^–1^ for *T. lutarioriparia* (Chen, 2020). Based on field observations in recent years, the area of the lake occupied by *P. australis* has gradually expanded, whereas the area occupied by *T. lutarioriparia* has gradually reduced. We hypothesize that common, widespread plant species, like *P. australis*, have a higher competitive ability than common endemic species like *T. lutarioriparia*. Therefore, to test the hypothesis, a field monitoring and manipulation experiment were conducted to compare the intensity, effect, and outcomes of competition between the two species.

## Materials and Methods

### Study Site

The Yangtze River is connected to Dongting Lake through three inlets (Songzikou, Taipingkou, and Ouchikou) and one outlet (Chenglingji). The Dongting Lake covers an area of 2,625 km^2^ (28°38′–29°45′ N, 111°40′–113°10′ E) and is divided into East, South, and West Dongting Lake. The study area has a subtropical monsoon climate, with an average annual temperature of 16.2–17.8°C and 259–277 frost-free days per year. Mean annual precipitation ranges from 1,200 to 1,415 mm, with the rainy season lasting from May to September. The average humidity is 80%, and the average evaporation is 1,270 mm. The annual mean wind speed is 2–3 m s^–1^ and the elevation is 28–35 m above sea level.

### Field Monitoring

#### Fixed Plot Selection

To compare the competitive ability between *T. lutarioriparia* and *P. australis*, in May 2018, five fixed plots comprising three mixed and two single communities were selected on the beach in the Dongting Lake wetlands. The plots were submerged due to flooding from May to September and exposed from October to April the following year. The fixed plot area was 5 m × 5 m, and the key characteristics of the plots are provided in Table 1.

**TABLE 1:** Numbers of *Triarrhena lutarioriparia* (*TL*) and *Phragmites australis* (*PA*) in 1 m^2^ area before and after flooding (BF and AF, respectively) in the five fixed communities and geographical coordinates of the communities in the East Dongting Lake wetlands in the field monitoring experiment.

	9:0	14:1		10:5		7:6		0:11
	*TL*	*TL*	*PA*	*TL*	*PA*	*TL*	*PA*	*PA*
BF	9	14	1	10	5	7	6	11
AF	8	6	0	5	3	5	4	9
Longitude	113°3′56.87	113°4′14.79″		113°5′7.78″		113°6′26.03″		113°7′44.28″
Latitude	29°24′52.12″	29°25′18.77″		29°25′23.75″		29°25′47.90″		29°26′20.40″

*The ratios 9:0, 14:1, 10:5, 7:6, and 0:11 are the ratios of individual numbers of TL to PA in a square meter.*

#### Fixed Plot Monitoring

In the fixed plots, the height and number of living individuals were recorded before flooding and after the floodwater had retreated. In the single communities, six individuals across a diagonal section of the plot were selected from each of the five plots. In the mixed communities, six plants, including three *T. lutarioriparia* plants and three *P. australis* plants, were selected in each of the five plots. The aboveground parts (leaves and stems) were collected and taken to the laboratory and dried to constant weight.

### Manipulation Experiment

#### Seed Collection and Germination

A manipulation experiment was performed to accurately control the initial ratio of *T. lutarioriparia* and *P. australis* in communities and continuously compare the competitive ability between the two species. In December 2017, seeds of *T. lutarioriparia* and *P. australis* were collected from the bottomland (29°15′15′′N, 112°49′20′′E) of the East Dongting Lake wetlands and brought back to the laboratory at Hunan Agricultural University. The mean weight of a thousand seeds was 0.35 ± 0.018 g (mean ± standard error) for *T. lutarioriparia* and 0.28 ± 0.004 g for *P. australis*. The mean seed length was 2.33 ± 0.034 mm for *T. lutarioriparia* and 2.03 ± 0.037 mm for *P. australis*. The mean seed width was 0.540 ± 0.014 mm for *T. lutarioriparia* and 0.57 ± 0.013 mm for *P. australis*. In March 2018, the seeds were sown in the field and germinated.

#### Seedling Transplantation

**FIGURE 1:**
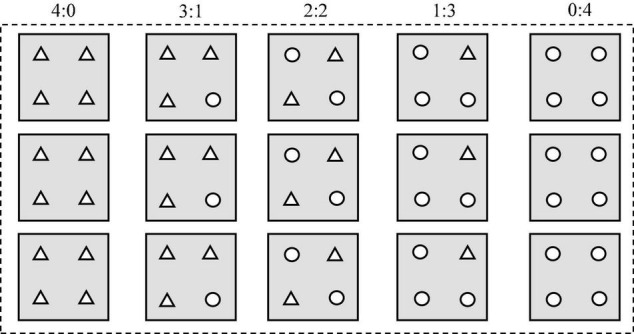
Planting patterns for the two species in each bucket in the manipulation experiment (: bucket, ^□^: basin, Δ: *Triarrhena lutarioriparia*, ^○^: *Phragmites australis*). The 4:0, 3:1, 2:2, 1:3, and 0:4 were the ratios of seedling numbers of *T. lutarioriparia* to *P. australis* in a basin, respectively.

In April 2018, seedlings with an aboveground height of 6–8 cm were transplanted into basins (12 cm in length, 12 cm in width, and 35 cm in height) filled up to 34 cm with soil (21 g kg^–1^ organic matter, 141 mg kg^–1^ exchangeable N, and 11.4 mg kg^–1^ exchangeable P). In brief, four seedlings were planted in each basin, and five planting patterns (the ratios of seedling numbers of *T. lutarioriparia* to *P. australis* were 4:0, 3:1, 2:2, 1:3, and 0:4, respectively) were used (Figure 1). For example, the pattern of 3:1 indicated that there were three *T. lutarioriparia* seedlings and one *P. australis* seedling in the basin. Red and white ropes were tied to the root base of each plant to distinguish between *T. lutarioriparia* and *P. australis* plants, respectively. There were 24 basins for each pattern, making a total of 120 basins. Fifteen basins were placed into a bucket (68 cm in length, 47 cm in width, and 41 cm in height) outdoors, and a total of eight buckets were used in the experiment (Figure 1). In total, twelve plants of each species were initially collected and the dry weights were measured. Following the transplantation of seedlings, water was added into the buckets until it reached the soil surface in the basin. This was the only time that water was added artificially into the bucket to simulate the natural environment.

#### Seedling Harvest

After transplanting the seedlings into basins, the plants were grown for 60, 120, and 180 days, and were then harvested. For a specific planting pattern, eight basins were selected for harvesting. The plants were dug out of the soil and carefully separated to maintain their integrity. The number of living plants, buds, and tillers for each species in the planting pattern was recorded. The dry biomass of the leaves, stems, roots, and buds was determined after drying at 80°C to a constant weight.

### Data Calculation and Analysis

#### Plant Growth Parameters

The survival percentage was calculated as the number of living plants divided by the initial number of plants.

The relative growth rate (*RGR*) was calculated using the following equation (Wang, 2013):


(1) $$R\,G\,R=\frac{l\,n\,Y_{2}-l\,n\,Y_{1}}{t_{2}-t_{1}}$$

where *Y*_1_ is biomass at harvest time *t*_1_, and *Y*_2_ is biomass at harvest time *t*_2_.

The relative elongation rate (*RER*) was calculated using the following equation (Wang, 2013):


(2) $$R\,E\,R=\frac{l\,n\,{\text{h}}_{2}-l\,n\,{\text{h}}_{1}}{{\text{t}}_{2}-{\text{t}}_{1}}$$

where *h*_1_ is plant height at harvest time *t*_1_, and *h*_2_ is plant height at harvest time *t*_2_.

#### Plant Competition Parameters

The relative competition intensity (*RCI*) can reflect plant competitive ability in three mixed communities (14:1, 10:5, and 7:6) in field monitoring, and the three mixed planting patterns (3:1, 2:2, and 1:3) in the manipulation experiment, and was calculated using the following equations (Grace, 1995):


(3) $$R\,C\,I_{i}=\left(Y_{i}-Y_{i\,j}\right)/Y_{i}$$

(4) $$R\,C\,I_{j}=\left(Y_{j}-Y_{j\,i}\right)/Y_{j}$$

where *Y*_*i*_ and *Y*_*j*_ are biomass of species *i* and species *j* in two single communities (9:0 and 0:11) in field monitoring, and two single planting patterns (4:0 and 0:4) in the manipulation experiment, respectively. *Y*_*ij*_ and *Y*_*ji*_ are biomass of species *i* and species *j* in mixed communities or planting patterns, respectively. *RCI*_*i*_ > *RCI*_*j*_ indicates that species *j* has a higher competitive ability than species *i*. *RCI*_*i*_ = *RCI*_*j*_ indicates that species *j* and *i* have the same competitive ability; and *RCI*_*i*_ < *RCI*_*j*_ indicates that species *i* has a higher competitive ability than species *j*. The known trend is that the lower the relative competition intensity (*RCI*), the greater the competitive ability.

The relative yield (*RY*) and the relative yield total (*RYT*) can reflect plant interspecific or intraspecific competition, and this was calculated using the following equations (Wang, 2013; Jiang et al., 2014):


(5) $$R\,Y_{i}=Y_{i\,j}/Y_{i}$$

(6) $$R\,Y_{j}=Y_{j\,i}/Y_{j}$$

(7) $$R\,Y\,T=\left(R\,Y_{\text{i}}+R\,Y_{j}\right)/2$$

where *RY* < 1 indicates that the plants have higher interspecific competition than intraspecific competition. *RY* = 1 indicates that the plants have the same interspecific and intraspecific competition, and *RY* > 1 indicates that plants have lower interspecific competition than intraspecific competition. *RYT* < 1 indicates that plants inhabit the same niches. *RYT* = 1 indicates that plants have partially the same niches. *RYT* > 1 indicates that plants have different niches.

The relative efficiency index (*REI*) and the expected relative efficiency index (*REI*_*exp*_) are used to predict dynamic changes in plant species in mixed communities or patterns, and were calculated using the following equations (Shipley, 1993; Grace, 1995):


(8) $$R\,E\,I=\left(\ln\,Y_{i\,j\,2}-\ln Y_{i\,j\,1}\right)-\left(\ln\,Y_{j\,i\,2}-\ln Y_{j\,i\,1}\right)$$

(9) $$R\,E\,I_{e\,x\,p}=\left(\ln\,Y_{i\,2}-\ln Y_{i\,1}\right)-\left(\ln\,Y_{j\,2}-\ln Y_{j\,1}\right)$$

where *Y*_*ij1*_ and *Y*_*ij2*_ are the biomass of species *i* in mixed communities or planting patterns at harvest times *t*_1_ and *t*_2_, respectively. *Y*_*ji1*_ and *Y*_*ji2*_ are the biomass of species *j* in mixed communities or planting patterns at harvest times *t*_1_ and *t*_2_, respectively. *Y*_*i1*_ and *Y*_*i2*_ are the biomass of species *i* in single communities or planting patterns at harvest times *t*_1_ and *t*_2_, respectively. *Y*_*j1*_ and *Y*_*j2*_ are the biomass of species *j* in single communities or planting patterns at harvest times *t*_1_ and *t*_2_, respectively. *REI* < *REI*_*exp*_ indicates that in mixed communities or planting patterns, species *j* will show increasing dominance over time. *REI* = *REI*_*exp*_ indicates that in mixed communities or planting patterns, species *j* will show stable dominance over time, and *REI* > *REI*_*exp*_ indicates that in mixed communities or planting patterns, species *j* will show decreasing dominance over time.

Multiple comparisons at the significance level of 0.05 were used to analyze the difference in biomass, RGR, relative elongation rate, tiller number, and bud numbers between different species and planting patterns. For these analyses, the IBM SPSS Statistics version 22 software (SPSS Inc., Chicago, IL, United States) was used.

## Results

### Survival Percentage

In the field monitoring experiment, the number of living individuals for both plant species decreased after flood retreat (Table 1). There was a pronounced difference in the survival percentage between the two plant species, except in the basins with a planting ratio of 14:1. With the increase in numbers, the survival percentage of *P. australis* increased, whereas that of *T. lutarioriparia* did not change.

In the manipulation experiment, both species had a decreased survival percentage during the experiment, except for the *P. australis* plants in basins with the planting patterns of 3:1, 1:3, and 0:4 (Table 2). At the same harvest time, *T. lutarioriparia* had a lower survival percentage than *P. australis*, except in the basins with the planting pattern of 1:3 on the 60th day.

**TABLE 2:** Survival percentages (%) of *Triarrhena lutarioriparia* (*TL*) and *Phragmites australis* (*PA*) on the 60th, 120th, and 180th days of plant transplantation in the manipulation experiment.

Day	4:0	3:1		2:2		1:3		0:4
	*TL*	*TL*	*PA*	*TL*	*PA*	*TL*	*PA*	*PA*
60	90.6	95.8	100.0	93.8	100.0	100.0	95.8	100.0
120	87.5	79.2	100.0	68.8	93.8	50.0	95.8	100.0
180	75.0	58.3	100.0	68.8	87.5	37.5	95.8	100.0

*The ratios 4:0, 3:1, 2:2, 1:3, and 0:4 are the planting patterns described in Figure 1.*

### Plant Biomass, Relative Growth Rate, and Relative Elongation Rate

**FIGURE 2:**
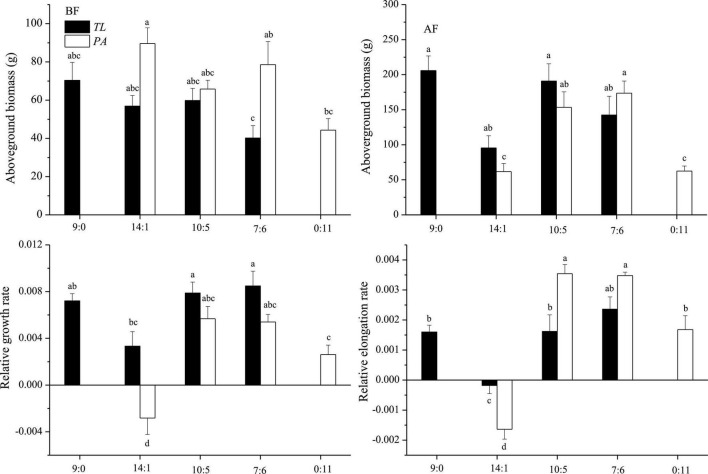
Aboveground biomass of *T. lutarioriparia* (*TL*) and *P. australis* (*PA*) before and after flooding (BF and AF, respectively), and their relative growth and elongation rates during the flooding period in the field monitoring experiment. The ratios 9:0, 14:1, 10:5, 7:6, and 0:11 are the ratios of individual numbers of *TL* to *PA* in a square meter. Different lowercase letters indicate significant differences among planting patterns at the 0.05 significance level.

In the field monitoring experiment, *T. lutarioriparia* had higher aboveground biomass than *P. australis* before flooding and lower aboveground biomass after flood retreat (Figure 2). During the flooding, *T. lutarioriparia* had a higher RGR and lower relative elongation rate compared to *P. australis*.

**FIGURE 3:**
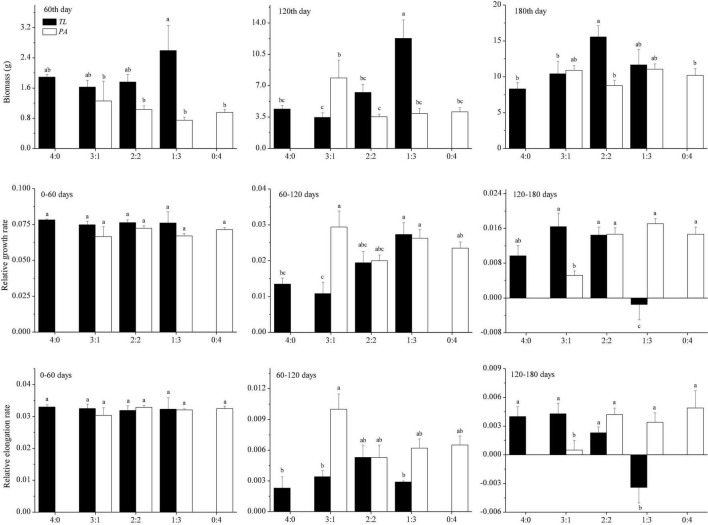
Biomass of *Triarrhena lutarioriparia* (*TL*) and *Phragmites australis* (*PA*) on the 60th, 120th, and 180th days of plant transplantation, and their relative growth and elongation rates in the periods of 0–60, 60–120, and 120–180 days in the manipulation experiment. The ratios 4:0, 3:1, 2:2, 1:3, and 0:4 are the planting patterns described in Figure 1. Different lowercase letters indicate significant differences among planting patterns at the 0.05 significance level.

In the manipulation experiment, *T. lutarioriparia* had higher biomass than *P. australis* in the early growth stage, whereas no difference in the biomass was observed between these two species in the later growth stage (Figure 3). The plant biomass showed different trends with the change in planting patterns. On the 60th day, there was no significant difference in the plant biomass between the two species for any of the four planting patterns. On the 120th day, the biomass of *T. lutarioriparia* was higher in basins with the 1:3 planting pattern than those with the other three patterns. The *P. australis* biomass was higher in basins with the 3:1 planting pattern than those with the 2:2 planting pattern. On the 180th day, the biomass of *T. lutarioriparia* was higher in the basins with the 2:2 planting pattern than those with the 4:0 and 3:1 planting patterns. However, there were no significant differences in the biomass of *P. australis* between the four planting patterns.

In the manipulation experiment, the relative growth and elongation rates were lower in *T. lutarioriparia* than in *P. australis* over 60–120 days, whereas they were not significantly different over 0–60 and 120–180 days. In the 0–60-day period, there were no significant differences in the relative growth and elongation rates between the four patterns in the two species. Over the 60–120-day period, the relative growth rate of *T. lutarioriparia* was lower in basins with the 3:1 planting pattern and higher in those with the 1:3 planting pattern, and that of *P. australis* was not significantly different in the basins for any of the four patterns. The relative elongation rate of either species did not differ between the four patterns. Over 120–180 days, the relative growth and elongation rates of *T. lutarioriparia* were lower in basins with the 1:3 planting pattern than in the basins with the other patterns, and those of *P. australis* were lower in basins with the 3:1 planting pattern than in the basins with the other patterns.

### Relative Competition Intensity and Relative Yield

In the field monitoring experiment, the relative competition intensity of the two plant species was different between two-time points (before and after flooding) and in three mixed communities (Table 3). In *T. lutarioriparia*, the flooding increased the relative competition intensity in the basins with the 14:1 and 10:5 patterns and decreased in the basins with the 7:6 pattern. In *P. australis*, flooding increased the relative competition intensity in the basins with the 14:1 and 7:6 patterns and decreased in the basins with the 10:5 pattern. The relative competition intensity of *T. lutarioriparia* was higher than that of *P. australis*. The relative yield of the two plant species also differed between the two-time points (before and after flooding) and the three mixed communities. The relative yield of *T. lutarioriparia* (≤1) was lower than that of *P. australis* (≥1). The relative yield total for the three mixed communities at the two-time points was > 1, indicating that the plants had different niches in the different communities.

**TABLE 3:** Relative competition intensity (*RCI*), relative yield (*RY*), and relative yield total (*RYT*) of *Triarrhena lutarioriparia* (*TL*) and *Phragmites australis* (*PA*) before and after flooding (BF and AF, respectively) in the field monitoring experiment.

Time	Communities	*RCI*		*RY*		*RYT*
		*TL*	*PA*	*TL*	*PA*	
BF	14:1	0.02	−0.53	0.98	1.53	1.26
	10:5	−0.03	−0.13	1.03	1.13	1.08
	7:6	0.31	−0.34	0.70	1.34	1.02
AF	14:1	0.53	0.01	0.47	0.99	0.73
	10:5	0.06	−1.45	0.94	2.46	1.70
	7:6	0.30	−1.78	0.70	2.78	1.74

*The ratios 14:1, 10:5, and 7:6 are the individual numbers of TL to PA in a square meter.*

In the manipulation experiment, the relative competition intensity of *T. lutarioriparia* decreased in basins with the 3:1 and 2:2 planting patterns and increased over time in those with the 1:3 planting pattern (Table 3). However, the relative competition intensity of *P. australis* decreased in the basins with the 3:1 and 1:3 planting patterns and increased in those with the 2:2 planting pattern. *T. lutarioriparia* had a lower relative competition intensity than *P. australis* in basins with the 1:3 planting pattern on the 60th day and in basins with the 2:2 planting pattern on the 120th day, and higher relative competition intensity than *P. australis* in those with the other planting patterns. The relative yield of *T. lutarioriparia* increased over time in basins with the 3:1, 2:2, and 1:3 planting patterns (Table 4). However, the relative yield of *P. australis* decreased in basins with the 2:2 planting pattern and increased over time in those with the 3:1 and 1:3 planting patterns. *T. lutarioriparia* had a lower relative yield than *P. australis* in basins with the 1:3 and 2:2 planting patterns on the 60th day and in basins with the 1:3 planting pattern on the 120th day, and a higher relative yield than *P. australis* did in the basins with the other patterns. The total relative yield for both plants in all three patterns and three harvest times was > 1, indicating that the plants had different niches in different planting patterns.

**TABLE 4:** Relative competition intensity (*RCI*), relative yield (*RY*), and relative yield total (*RYT*) of *Triarrhena lutarioriparia* (*TL*) and *Phragmites australis* (*PA*) on the 60th, 120th, and 180th days of plant transplantation in the manipulation experiment.

Days	Patterns	*RCI*		*RY*		*RYT*
		*TL*	*PA*	*TL*	*PA*	
60	3:1	0.14	−0.31*	0.86^e^	1.31^a^	1.09
	2:2	0.07	−0.08*	0.93^e^	1.08^a^	1.01
	1:3	−0.37*	0.22	1.37^a^	0.78^e^	1.08
120	3:1	0.21	−0.92*	0.79^e^	1.92^a^	1.36
	2:2	−0.42*	0.14	1.42^a^	0.86^e^	1.14
	1:3	−1.80 *	0.05	2.80^a^	0.95^e^	1.88
180	3:1	−0.25*	−0.07	1.25^a^	1.07^a^	1.16
	2:2	−0.87*	0.14	1.87^a^	0.86^e^	1.37
	1:3	−0.40*	−0.08	1.40^a^	1.08^a^	1.24

The ratios 4:0, 3:1, 2:2, 1:3, and 0:4 are the planting patterns described in Figure 1. *Indicates that the competition ability was strong.

*^a^Indicates that intraspecific competition was higher than the interspecific competition.*

*^e^Indicates that intraspecific competition was lower than interspecific competition.*

### Relative Efficiency Index and Expected Relative Efficiency Index

In the field monitoring experiment, the relative efficiency index was lower than expected, indicating that *P. australis* would be the dominant species over time (Table 5). In the period of 0–60 days, the relative efficiency index was lower than expected in basins with the 3:1 and 2:2 patterns, and higher than expected in the basins with the 1:3 pattern. This indicates that in this period, *P. australis* would be the dominant species in basins with the 3:1 and 2:2 patterns, and *T. lutarioriparia* would be the dominant species in those with the 1:3 pattern (Table 6). In the period of 60–120 days, *P. australis* would be the dominant species in basins with the 3:1 pattern, and *T. lutarioriparia* would be the dominant species in those with the 2:2 and 1:3 patterns. In the period of 120–180 days, *T. lutarioriparia* would be the dominant species in basins with the 3:1 and 2:2 patterns, and *P. australis* would be the dominant species in those with the 1:3 pattern. During the whole experimental period (0–180 days), *T. lutarioriparia* would be the dominant species in basins with mixed patterns.

**TABLE 5:** Relative efficiency index (*REI*) and expected relative efficiency index (*REI*_*exp*_) during flooding in the field monitoring experiment.

	***REI***		***REI***_***exp***_
14:1	10:5	7:6	Single communities
0.89^P^	0.31^P^	0.47^P^	1.19

*The ratios 14:1, 10:5, and 7:6 are the individual numbers of TL to PA in a square meter. ^P^Indicates that Phragmites australis would be the dominant species.*

**TABLE 6:** Relative efficiency index (*REI*) and expected relative efficiency index (*REI*_*exp*_) in the periods of 0–60, 60–120, 120–180, and 0–180 days in the manipulation experiment.

Days	*REI*			*REI*_*exp*_
	3:1	2:2	1:3	Single planting
0–60	−0.03^P^	0.25^P^	0.96^T^	0.39
60–120	−1.08^P^	0.04^T^	−0.10^T^	−0.61
120–180	0.78^T^	0.01^T^	−1.09^P^	−0.28
0–180	−0.33^T^	0.29^T^	−0.23^T^	−0.49

*The ratios 3:1, 2:2, and 1:3 are the planting patterns described in Figure 1.*

*^T^Indicates that Triarrhena lutarioriparia would be the dominant species.*

*^P^Indicates that Phragmites australis would be the dominant species.*

## Discussion

### Growth Performance

Plant survival is one of the most direct reflections of plant adaptation, especially under environmental stress. In the field monitoring experiment, both *P. australis* and *T. lutarioriparia* showed a decreased survival percentage during flooding, which did not differ between the species. In the Dongting Lake wetlands, deeper submergence led to the death of many wetland plants, such as *Salix triandroides* and *Carex tristachya* (Ding et al., 2017, 2019). However, compared with deeper submergence in the field monitoring, low depth submergence in the manipulation experiment did not lead to variation in the survival percentage of *P. australis* and decreased the survival percentage of *T. lutarioriparia*. This indicated that *P. australis* exhibited a higher survival percentage than *T. lutarioriparia*, suggesting that *P. australis*, a widespread species, is more adaptable to environmental changes than *T. lutarioriparia*, an endemic species (Eller et al., 2017).

Plant growth can also reflect plant adaptation to environmental stress. In the field monitoring experiment, *P. australis* had higher aboveground biomass before flooding and a higher relative elongation rate during flooding, whereas *T. lutarioriparia* had higher aboveground biomass after flooding and a higher relative growth rate during flooding. However, in the manipulation experiment, the plant biomass, relative growth rate, and relative elongation rate were not different between the two species. These differences in plant growth between the field monitoring and the manipulation experiment may be attributed to the difference in water conditions. In the field experiment, a 5-month of flooding caused different response patterns in the plants: the well-developed stem porosity in *T. lutarioriparia* led to increased oxygen transmission from the aboveground parts to the belowground parts and resulted in increased growth, whereas the relatively lower stem porosity in *P. australis* forced rapid vertical stem elongation so that stems were able to reach the water surface and access oxygen from the air (Jackson, 2008; Ding et al., 2017). In the manipulation experiment, the two plants showed similar growth characteristics because of the absence of flooding stress.

### Competition Performance

Plant competition can be determined by the competitive intensity and is associated with community characteristics, such as biomass, density, and proportion (Zhang and van Kleunen, 2019). The competition performance of *P. australis* and *T. lutarioriparia* also differed between the field monitoring and the manipulation experiment. Overall, *P. australis* showed a higher competitive ability than *T. lutarioriparia* in the field monitoring and a lower competitive ability than *T. lutarioriparia* in the manipulation experiment. Similar to the reasons for differences in plant growth, seasonal flooding reduced the competitive ability of *T. lutarioriparia* and improved the competitive ability of *P. australis*.

Plant competitive ability includes the ability of intraspecific and interspecific competition (Connolly, 1987; Weigelt and Jolliffe, 2003). In the field monitoring experiment, intraspecific competition was higher in *P. australis*, and interspecific competition was higher in *T. lutarioriparia*. However, over time, the competition changed from intraspecific to interspecific in *P. australis* and from intraspecific to interspecific in *T. lutarioriparia*. Competition often leads to species occupying different ecological niches (Sophie and Sylvie, 2012). The total relative yield for the field monitoring and the manipulation experiments was > 1, indicating that the two plant species had different niches. It was found that *P. australis* would become the dominant species in the field monitoring experiment and that *T. lutarioriparia* would become the dominant species in the manipulation experiment.

The present study showed that the competitive ability of plant species varied depending on environmental conditions. In the submerged habitat, the competitive ability of *P. australis* was higher than that of *T. lutarioriparia*, whereas the opposite was true in the non-submerged habitat. In the Dongting Lake wetlands, the 5-month flooding period increased the dominance of *P. australis*, and the 7-month non-flooding period increased the dominance of *T. lutarioriparia*. However, global climate change has led to long-term high-density flooding in recent years. For instance, in 2020, water in the wetlands remained at a high level for 38 consecutive days. This has led to area expansion of *P. australis* in the Dongting Lake wetlands. Therefore, niche differences between these two species enable their coexistence in the Dongting Lake wetlands under the influence of seasonal flooding.

## Data Availability Statement

The original contributions presented in the study are included in the article/supplementary material, further inquiries can be directed to the corresponding author.

## Author Contributions

YL designed the study. YD and ZP performed the field monitoring experiment. QZ, FP, and LX conducted the control experiment. YD and YL wrote the manuscript and other authors revised it. All authors contributed to the article and approved the submitted version.

## Conflict of Interest

The authors declare that the research was conducted in the absence of any commercial or financial relationships that could be construed as a potential conflict of interest.

## Publisher’s Note

All claims expressed in this article are solely those of the authors and do not necessarily represent those of their affiliated organizations, or those of the publisher, the editors and the reviewers. Any product that may be evaluated in this article, or claim that may be made by its manufacturer, is not guaranteed or endorsed by the publisher.
